# Aqueous extract of saffron administration along with amygdala deep brain stimulation promoted alleviation of symptoms in post-traumatic stress disorder (PTSD) in rats

**Published:** 2018

**Authors:** Mina Mokhtari Hashtjini, Gila Pirzad Jahromi, Gholam Hossein Meftahi, Davoud esmaeili, Danial Javidnazar

**Affiliations:** 1 *Neuroscience Research Centre, Baqiyatallah University of Medical Sciences, Tehran, Iran *; 2 *Electrophysiology Research Center, Neuroscience Institute, Tehran University of Medical Sciences, Tehran, Iran *; 3 *Applied Microbiology Research Center, and Microbiology Department, Baqiyatallah University of Medical Sciences, Tehran, Iran*

**Keywords:** Deep brain stimulation, Saffron, Post-traumatic stress disorder, Contextual fear conditioning, Corticosterone, C-Fos protein

## Abstract

**Objective:**

Post-traumatic stress disorder (PTSD) as one of the most devastating kinds of anxiety disorders, is the consequence of a traumatic event. *Crocus sativus* L., commonly known as saffron have been traditionally used for treatment of stress and anxiety. In this study, we evaluated the effects of peripheral administration of saffron, along with deep brain stimulation (DBS) in a post-traumatic stress disorder (PTSD) model caused by contextual fear conditioning (electrical foot shock chamber) in male Wistar rats.

**Materials and Methods:**

rats (220-250 g) were divided into 7 groups (n=8) and underwent stereotactic surgery for implantation of the electrodes in the right-baso lateral of the amygdala (BLA). After 7 days, some animals received the foot shock, followed by another 7-day treatment (DBS treatment or combination treatment by saffron 5 mg/kg (i.p)) then freezing behavior as a predicted response in the absence of the foot shock (re-exposure time) and general anxiety were measured using elevated plus maze test. Serum corticosterone level and amygdala c-Fos protein expression were assessed using ELISA and Western blot analysis, respectively.

**Results:**

DBS treatment and the combination therapy of saffron (5 mg/kg (I.P)) with DBS significantly (p<0.001) increased serum corticosterone levels. Also both treatments could significantly (p<0.001) reduce c-Fos protein expression and freezing behaviors time. However, DBS treatment had no effect on the general anxiety in rats with PTSD. On the other hand, combination therapy significantly (p<0.001) reduced anxiety behavior in rats with PTSD.

**Conclusion:**

These results might show the potential of this combination therapy for treatment of treatment-resistant PTSD patients.

## Introduction

Post-traumatic stress disorder (PTSD) which occurs as a consequence of a completely traumatic event such as intense fear, is considered an important kind of anxious disorder (Langevin et al., 2010 ▶). Also, PTSD is a devitalizing disorder coupled with functional impairments and physical and some mental health conditions such as depression resulting in a higher risk of suicide. Treatment of PTSD is a really challenging issue, and medicine such as antidepressants, anxiolytic drugs, β-adrenergic antagonists, opiates, and cortisol have shown controversial results (Kar, 2011 ▶).

 Studies based on functional magnetic resonance imaging (fMRI) in PTSD patients revealed a significant hyperactivity in the amygdala (Langevin et al., 2010 ▶). Additionally, there is a defect in the fear extinction recall in PTSD patients, which is a consequence of a referenced failure of the amygdala’s basolateral nucleus (BLn) (Yehuda, 2009 ▶; Franzini et al., 2013 ▶; Maren et al., 2013 ▶; Stidd et al., 2013 ▶; Marin et al., 2014 ▶) . Therefore, modifying BLn amygdala activity by surgical therapies such as deep brain stimulation (DBS) that transmits a high-frequency current by an electrode through subcortical structures, seems appropriate (Rosa et al., 2012 ▶; Marin et al., 2014 ▶). During past decades, DBS application have resulted in significant progress in modifying some psychiatric conditions such as depression and obsessive-compulsive disorder. In fact, DBS treatment could considerably alleviate symptoms in PTSD patients or animal models (de Koning et al., 2013 ▶). Meanwhile, some studies focused on DBS mechanism and suggested different hypotheses for DBS effect which are not deterministic (McIntyre et al., 2004 ▶; Mayberg et al., 2005 ▶; Yu, 2008 ▶; Langevin, et al., 2013 ▶; Marin et al., 2014 ▶). 

Indeed, recent studies indicate that in spite of all advantages of DBS in alleviating some PTSD symptoms such as avoidance behavior, it has no effect on the general anxiety behavior caused by PTSD (Stidd et al., 2013 ▶).

*Crocus sativus* L., commonly known as saffron, as a bulbous perennial member of the Iris family (Iridaceae) is famous for its golden color and strong flavoring activity and has been traditionally used as a food ingredient (Hosseinzadeh , 2009 ▶; Hosseinzadeh , 2010 ▶; Khazdair , 2015 ▶).

One of saffron’s principal coloring pigments is crocin, which is easily soluble in water. Saffron aqueous extract and its active constituents have shown effects on a number of different neural conidtions, which resulted in healing memory impairments in rodents and antidepressant effects in human (Noorbala et al., 2005 ▶; Georgiadou et al., 2012 ▶; Mokhtari-Zaer, 2015 ▶). Although some studies have evaluated the effect of aqueous extract of saffron in PTSD (Sahraei et al., 2012 ▶), robust data confirming the certain effect of saffron aqueous extract on PTSD and it behavioral symptoms, is still lacking. 

PTSD underlying mechanisms are similar to those of the fear acquisition and extinction; hence, the fear conditioning seems to be an appropriate model for PTSD (Delgado et al., 2006 ▶; Milad et al., 2006 ▶; Yehuda, 2007 ▶). Our animal model was a contextual fear conditioning which exactly mimics PTSD which is followed by an avoidance behavior such as freezing. Notably, in order to investigate the therapeutic effects of co-administration of DBS and aqueous extract of saffron, we examined corticosterone levels in sera of rats as it possesses an important role in the formation of emotional fear and conditional fear in PTSD (De Kloet et al., 2006 ▶). In addition, another contributing factor that was investigated in this study, was the level of c-Fos expression, especially in the amygdala, which plays a significant role in learning memory of fear. Moreover, it should be noted that the level of c-Fos protein expression in the amygdala reflects neuronal activity (Frances Davies et al., 2003 ▶).

With the aim of finding a novel and effective treatment for PTSD symptoms, the present study was designed to evaluated the co-administration of DBS and aqueous extract of saffron.

## Materials and Methods

**Animal**

Male Wistar rats (220-250 g) were obtained from Pasteur Institute, Tehran, Iran. The rats were housed individually during the experiment with a light/dark cycle of 12 hr/12 hr. Water and food were freely available. The experimental protocol was reviewed and approved by the Institutional Committee of Animal Care and Use, Tehran University of Medical Sciences, Tehran, Iran. Notably, experiments were conducted during daylight hours, under the standard conditions. At the end, all animals were sacrificed and the brain were quickly removed.

**Experimental procedure**

All the animals were randomly divided into 7 groups (each contained 8 rats):

1. Intact animals

2. Sham (rats that underwent surgery circumstance without electrode implantation)

3. Negative Control (rats with PTSD that did not received treatment)

4. Positive control (rats with PTSD+ that received saline) as solvent medication

5. Saffron treatment (rats with PTSD that received aqueous extract of saffron) 

6. DBS-treatment (rats with PTSD (surgery) that received DBS)

7. Combination treatment (rats with PTSD (surgery) that received both DBS and aqueous extract of saffron)

**Figure 1:**
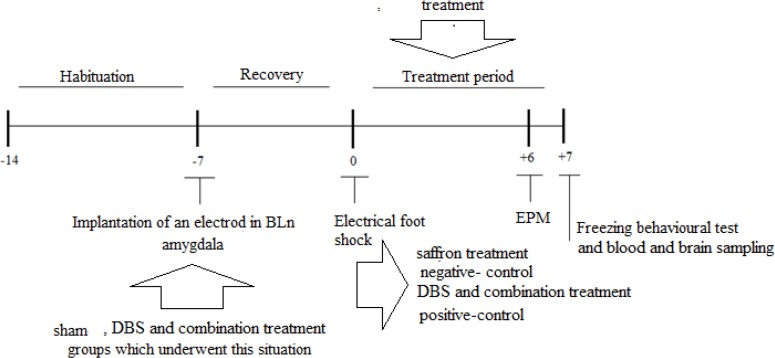
Experiment design and time schedule

Evaluation of rats was done after a 7-day post-surgery recovery period (Figure 1). 

**Surgical method**

Following the adaptation period, the PTSD groups underwent surgery for settlement of a single intracranial electrode in the right amygdala. The electrode was a Teflon-plated stainless steel wire (8 mm in length), fitted to the desired core to the surface of the head. Then, anesthesia was induced using ketamine (30 mg/kg, i.p.) and xylazine (15 mg/kg, i.p.). Rats’ head was held by a stereotaxic frame and the hair of the top of the head was removed and cleaned with iodine and ethanol; next, a cut line was made on the exact modeling of the cranium following the coordinates given in the Paxinos and Watson Atlas (1987). The certain point (lateral 4.8mm, anterior-posterior -2.5mm, ventral-dorsal 7.4mm) on right amygdala was secured after making a small hole by a dental drill at the exact point. The electrode which was attached to the plastic connector of stereotaxic arm was gently inserted in the Bln. The connector was stayed on the surface of the skin using dental cement and 4 screws and the arms of the stereotaxic device were released. Following the operation, the animals were given 7 days to rest. Buprenorphine (0.1 - 0.5 mg/kg, i.p.) was administered daily for postoperative analgesia for 3 days.

**Contextual Fear conditioning (CFC), inescapable foot shock**

CFC training and behavioral testing took place in a rodent observation cage (30×37×25 cm) placed inside a soundproof chamber. The side walls of the cage were made of stainless steel and the back walls and doors were made of clear Plexiglas. The floor contained 20 steel rods connected to a shock generator (Sahraei et al., 2012 ▶). Each observation was cage cleaned with an ethanol 90% solution before and after each test. On the conditioning day, the rats were individually transferred to the cage and after 15 min of acclimatization, they received ten foot shocks (unconditional stimuli) each one for 1 sec with 2 mA intensity, with 30 sec intervals (Cordero et al., 2003 ▶, Langevin et al., 2013 ▶). After 10 min, the rats were removed from the cage. For the conditioning test after 7 days (retention), the rats were brought back to the cage (contextual fear conditioning) in the absence of the electrical foot shock and stayed there for almost 8 min. A camera recorded the behavior during both training and testing, the time spent by each rat either freezing or active, was measured by a blind researcher. Freezing as a behavioral marker was interpreted an immobile posture except for respiration movement. Behavior was scored for the testing part, the behavior evaluated during the 8-min re-exposure to the context and scores for each part was recorded individually (Sahraei et al., 2012 ▶).

**Plant material**

The saffron used in this experiment was donated by Talakaran‐E‐Mazraeh Agricultural Co. The plant was authenticated by M. Kamalinejad (Department of Pharmacognosy, Faculty of Pharmacy, Shahid Beheshti University of Medical Sciences, Tehran, Iran) and a voucher specimen (No. P‐408) was deposited at the herbarium of Department of Pharmacognosy, Faculty of Pharmacy, Shahid Beheshti University of Medical Sciences, Tehran, Iran. The part of *Crocus sativus* that is used as an additive and an herbal medicine is the stigma. The stigma’s extract was prepared as follows: Saffron powder (100 g) was mixed with distilled water (1:50 w/v) and left on a shaking incubator at 8 °C for 48 hr. The solution was centrifuged (at 4000 g for 10 min). The yielded supernatant was retained and the sediment was suspended in the half amount (500ml) of mentioned distilled water and placed on the shaking incubator for another 24 hr. The centrifugation was repeated again and the resulting supernatant was separated and stored at −20 °C in a freezer. Evaporation of solvent was performed by freeze drying.

The yield of extraction was 25 g of dried powder for 100 g of the dry stigma for 25% aqueous extract. The extract was dissolved in normal saline and was immediately administered intraperitoneally (i.p.) at the dose of 5mg/kg to the rats (Halataei, Khosravi et al., 2011 ▶; Sahraei, Fatahi et al., 2012 ▶). 

**Quantification of crocin in saffron extract**

Crocin quantification in saffron aqueous extract was performed according to a previously described method with modifications (Hosseinzadeh and Noraei 2009 ▶). For the determination of crocins and safranal in saffron, samples were extracted with 8 mL of degassed methanol and sonicated for 1 hr and then stored overnight. The whole process was carried out in darkness at room temperature. Samples were removed from darkness, sonicated for one more hour, and brought to volume with previously degassed methanol. Each extracted sample was filtered using a 1-mL Tuberculin syringe and a 0.20 mm filter tip into an HPLC vial for HPLC analysis. A 25-mL sample was then injected into an HPLC system coupled with a diode array detector.

 Our data showed that the aqueous extract contained 22.5% crocin (Hosseinzadeh , 2009 ▶; Halataei et al., 2011 ▶). The extract doses used in these experiments were standardized according to their crocin content.

**Deep brain stimulation (DBS)**

 High-frequency DBS treatment started the first day after the contextual fear conditioning. All the animals of DBS groups were kept in individual cages during DBS. The external pulse generator was attached to the intracranial electrode with the plastic connector. There was enough wire to allow the animal to move freely in the cage. The DBS current was flowed for 1hr through the implanted electrodes constantly for 7 days for each rat. The current setting involved monopolar, 120 ms pulse width, 160 Hz frequency and 2.5 volts (Langevin et al., 2013 ▶). The control group rats were also connected to the pulse generator under the same setting, but received no pulse. 

**Elevated plus maze (EPM) test**

 The apparatus is a cross-shaped maze with 4 wooden arms; each arm was 90 cm away from the common center and the two opposite arms were open to the environment and the other opposite arms were surrounded by 40-cm walls except the way to the center. This maze is usually employed for evaluation of anxiety in rats (Sahraei et al., 2012 ▶). Depending on the time spent in the open arms within a 5-min period, the level of anxiety is examined. First, rats were placed in the center of the cross with the head facing one of the open arms. A blind examiner recorded movement factors. Other important factors were open arm entries (OAE %) and the time spent in open arm (OAT %) defined.

Corticosterone measurement

 To analyze corticosterone level in the serum of rats with PTSD and non-PTSD, approximately 2 ml blood was obtained from the orbital sinus of the rats’ eye. Consequently, the blood samples were centrifuged at 3500 rpm for 5 min at room temperature and the serum was stored at -70°C. Corticosterone level was measured using an ELISA DRG Corticosterone kit (Cat. No. EIA 4164).

**C-Fos expression assessment using western-blot analysis**

 c-Fos activation was evaluated by western blot as previously described. All rats were sacrificed at the end of the experiment and the whole brain tissue was immediately removed and carefully transferred from nitrogen tank to the storage place -80 °C). Approximately, a neat piece of Bln dissected from the brain (almost 1g) and placed in 1ml or 400µl of ice-cold SDS lysis buffer (2% SDS, 0.3% DTT, 10% glycerol in 40 mM Tris-cl; pH 6.8). Consequently, the samples were homogenized by ultrasonic disruption, and the obtained solution was heated to 85 °C for 8 min, then centrifuged at 12000 g for 12 min). The protein content were then separated from the pelleted debris. Total protein segments were separated by SDS-PAGE gel on Bio-Rad system and immunoblotted. Subsequently, semidry electron- transfer to nitrocellulose membranes was done (0.2-µm pore size) and then blots were incubated with the blocking solution, 5% BSA in Tris- buffered saline containing 0.1% Tween 20, overnight at 4 °C while being gently shaken. Next, blots were incubated in primary antibody solution (Anti-c-Fos antibody - ChIP Grade, Rabbit polyclonal to c-Fos - ChIP Grade, ab-cam ab7963. 1:200) which was dissolved in TBS-t, for 2 hr at 4 °C while being gently shaken. Afterward, blots were washed three times for 15 min with TBS-t and incubated for 1 hr at 1:10000 dilution of a secondary antibody (Dako REAL EnVision, Rabbit/Mouse, HRP conjugated) which was dissolved in TBS-t. Afterwards, membranes were washed three times with TBS-t, each time for 5 min. Finally, diaminobenzidine (DAB) which is a common substrate for horseradish peroxidase (HRP)- conjugated with secondary antibody, was used for visualizing the blots. For quantification, c-Fos band (as the examined protein) was normalized against beta-actin band (as the housekeeping protein), and densitometry values for each experimental group was calculated by using Image J software and reported as mean±S.E.M.

**Tissue confirmation**

To confirm that the electrode was correctly placed in the BLn amygdala, a cannula was implanted in BLn position and Trypan blue (10µg/per rat) was injected thorough the cannula using a Hamilton syringe. After injection, the animal was sacrificed and using the brain slides, the correct location of the cannula in the BLn area was confirmed.

**Statistical analysis**

The quantitative findings were presented as mean±SEM. One-way analysis of variance and complementary Tukey test were used to compare intergroup means. A p-value<0.05 was considered significant.

## Results

**Freezing behavior time**

**Figure 2:**
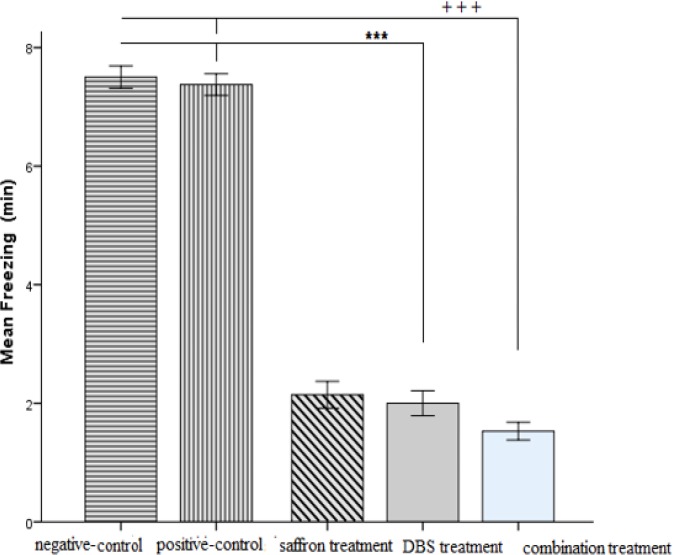
Changes in the freezing time; the exploratory behavior assessed 7-days post-shock in six groups at the re-exposure time in the context. ^***^p<0.001 and ^+++^p<0.001 indicate significant difference as compared to positive and negative control groups

The quantitative findings were presented as mean±SEM. Recorded mean time of the freezing behavior in the negative-control group indicated a significant (7.5±0.53, p<0.001) rise in the freezing time compared to the treatment groups (DBS treatment, saffron treatment and combination treatment). There were (p<0.001) significant differences among DBS treatment group (2.00±0.20) compared to negative-control (7.5±0.53, p<0.001) and positive-control (7.3±0.1, p<0.001) groups. Although there were significant (p<0.001) differences between combination treatment (1.5±0.1) and negative-control (7.5±0.53, p<0.001) and positive-control (7.3±0.1, p<0.001) groups. Moreover there were significant differences between saffron treatment group (2.1±0.2, p<0.001) and the negative and positive control groups (Figure 2). 

**Plasma corticosterone blood level**

The quantitative findings were presented as mean±SEM .Our data showed that the negative-control group had a significant reduction in serum corticosterone level (38.68±0.7, p<0.001) compared to both intact (76.96±2.83 p<0.001) and sham-operated groups (77.78±4.33, p<0.001). Meanwhile, both treatment groups (DBS treatment (60.12±1.41, p<0.001) and combination treatment (73.15±3.8, p<0.001)) and saffron-treated group (57.02±1.2, p<0.001) showed significant changes compared to the negative-control group (38.68±0.7, p<0.001) and positive control (43.2±1.7, p<0.001). 

**Figure 3:**
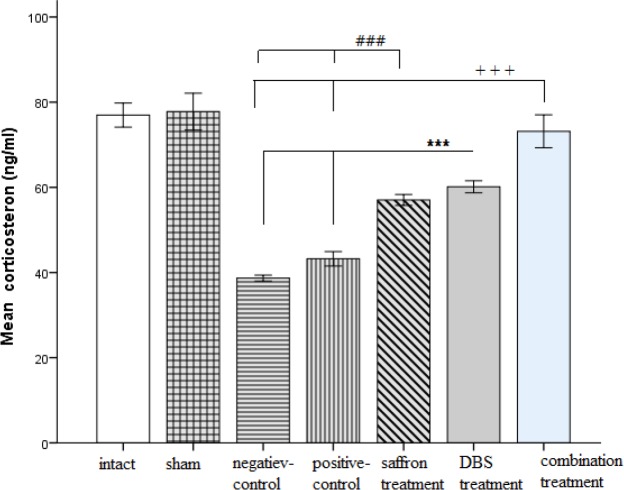
Serum corticosterone level presented after the re-exposure time in the groups. ***p<0.001 and +++p<0.001, ###p<0.001 indicate significant difference compared to control groups

Moreover, the combination treatment group indicated an exact amount of blood serum hormone as well as the intact group, despite the DBS treatment group (Figure3).

**c-Fos protein expression**

**Figure 4:**
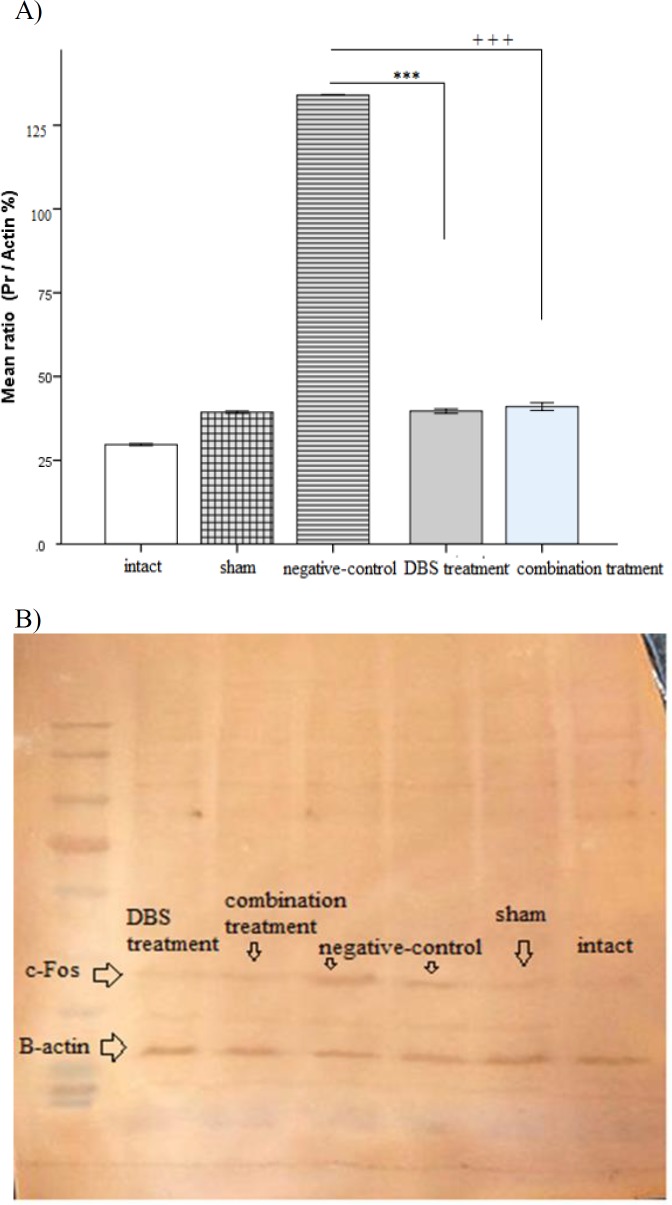
A) The amygdala c-Fos expression presented after the re-exposure time in the groups. ***p<0.001 and +++p<0.001 show significant differences as compared to negative control group. B) Western-blot analysis of c-Fos protein expression. There was a significant difference among group’s bands which declared the effect of DBS treatment

The quantitative findings were presented as mean±SEM. Western blot analysis indicated that negative-control group (1.34±0.00, p<0.001) had a significant rise in the amygdala c-Fos protein expression compared to both intact (0.29±0.005 p<0.001) and sham groups (0.39±0.005, p<0.001). On the other hand, the DBS treatment group (0.39±0.11, p<0.001) and the combination treatment group (0.41±0.02, p<0.001) showed no significant change compared to the intact and sham group; however, the negative-control group indicated a remarkable increase compared to both treatment groups (p<0.001) (Figures 4 A and B). 

**Anxiety-like behavior results**

**Figure 5:**
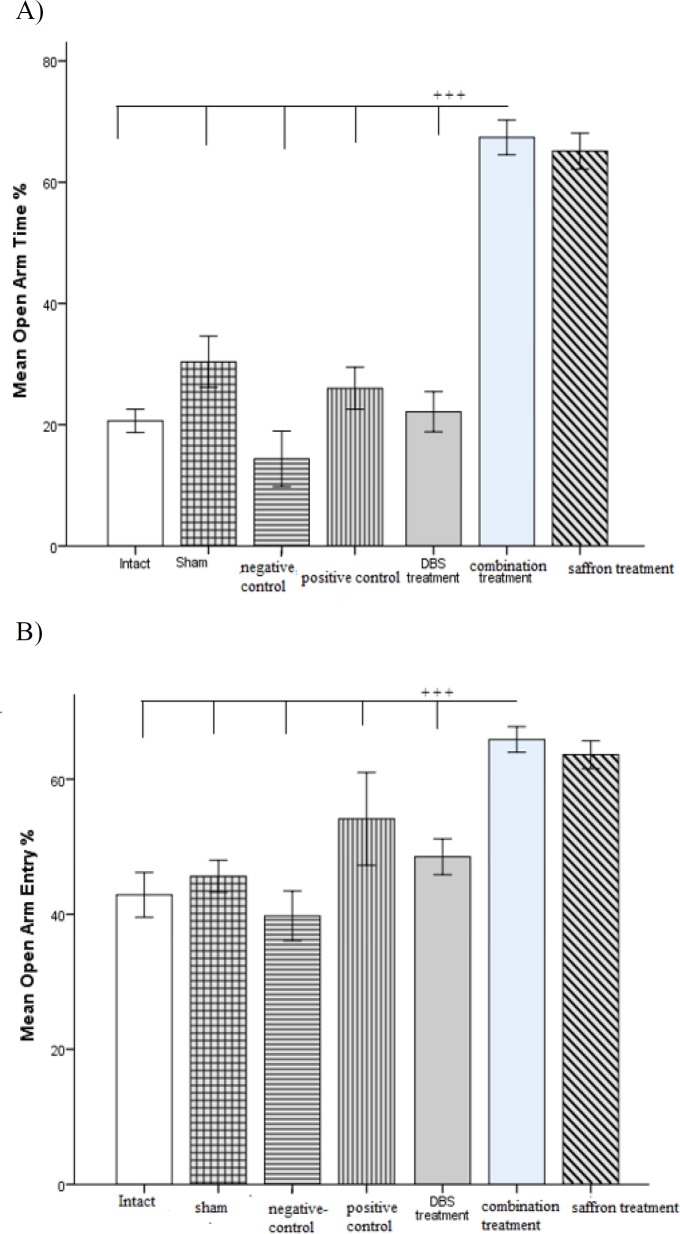
A) Changes in general anxiety as measured by EPM; Mean OAT (A) of EPM are shown for all the groups. ^+++^p<0.001 indicates significant difference among groups. B) Changes in general anxiety as measured by EPM; Mean OAE (B) of EPM are shown for all the groups. ^+++^p<0.001 indicates significant difference among groups

General anxiety-like behavior was evaluated by EPM test, on day 6 post-shock. The combination treatment group and saffron-treated group, both indicated remarkable changes in general anxiety-like behavior. There is a significant (p<0.001) difference between the combination treatment group and others in terms of open arm time and open arm entry ratio, which indicated the effect of saffron on anxious behavior (Figures 5 A and B).

## Discussion

The purpose of this study was to examine the effects of concurrent treatment with DBS and saffron aqueous extract because of its long-standing traditional use in the indigenous medicine of many countries (Hosseinzadeh , 2010 ▶) which were also shown by pervious findings. In order to investigate the effect of the combination of aqueous extract of saffron and DBS, a conditional fear model as a similar model to PTSD, was used. PTSD model that was chosen for this experiment reduced the levels of corticosterone in the blood; also, it increased the expression of c-Fos in the BLn amygdala. Consequently, the above-mentioned changes led to increased avoidance behavior and anxiety in rats with PTSD, which showed an increase in freezing time and anxious behavior. 

DBS treatment was able to noticeably increase the levels of corticosterone in serum of rats with PTSD. Moreover, overexpression of brain c-Fos protein was profoundly reduced in the DBS treatment group. Although, DBS significantly reduced the freezing behavior and alleviate PTSD symptoms, it had no effect on the anxiety behavior. 

In contrast, treatment with aqueous extract of saffron in the absence of DBS (called as saffron treatment group), improved the symptoms in rats with PTSD (i.e. increased the level of corticosterone or reduced the freezing behavior compared to the untreated groups). Additionally, unlike the DBS treatment, monotherapy with aqueous extract of saffron improved the anxious behavior.

Based on the data obtained, combination therapy increased corticosterone hormone levels and restored its levels to that of the intact group, compared with other therapeutic groups. Although DBS and monotherapy with saffron alone could increase the level of corticosterone compared to the rats with PTSD, there was a significant gap between the amount of serum hormone in the DBS treatment and saffron treatment compared with the intact group. It seems that the combination of these two treatments (as combination treatment) filled this gap.

 In addition, combination treatment significantly reduced both c-Fos protein expression and the freezing behavior time. Although the data do not show a significant difference among these two treatment groups, this does not mean that the combination treatment method is not effective. Since the improvement in the symptoms of rats with PTSD due to this method of combination treatment explained the benefit of the combination therapy as well as DBS treatment. In addition, the benefit of the combination treatment plus to the normal level of corticosterone is the improvement in the anxiety behavior in PTSD rats.

Previously, findings indicated that DBS stimulation modulates pathological network activity that might be a mechanism of the DBS treatment (McIntyre et al., 2004 ▶). Consistent with our results, DBS might lead to decreased activity of the amygdala and the mesocorticolimbic system, followed by reduction of cerebral dopamine and consequently dopamine-dependent behaviors such as freezing behavior in this case (Kvetnansky et al., 2009 ▶; Schiller et al., 2010 ▶). However, no significant changes observed in general trauma independent anxiety measured by elevated plus maze test in the DBS treatment group in this study. In fact, animals with less anxiety spent more time in the open arms. Therefore, it seems that DBS by modulating mesocorticolimbic system via amygdala connections might be more effective in avoidance behavior than anxiety behavior in rats with PTSD.

Saffron extract contains a notable amount of crocin may be able to inhibit PTSD (Hosseinzadeh , 2009 ▶; Hosseinzadeh , 2010 ▶; Halataei et al., 2011 ▶). Additionally, crocin as a main constituent of saffron extract has an affinity to inhibit glutamate N-methyl-D-aspartate (NMDA) receptor in the central nervous system; which appears to be the certain effect of saffron (Sahraei et al., 2012 ▶). Moreover, suppression of the glutamate NMDA receptors might lead to decreased activity of the mesocorticolimbic system, followed by reduction of cerebral dopamine and consequently the dopamine-dependent behaviors such as freezing behavior in this case (Noorbala et al., 2005 ▶, Sahraei et al., 2012 ▶). The present results indicate that treatment with aqueous extract of saffron (*Crocus sativus* L.) induces anxiolytic-like effects in rats. Based on the present results, it could be concluded that crocin as a main constituent of saffron might reduce the anxiety in animals exposed to the context- fear conditioning (PTSD model). The pharmacological mechanism(s) that might account for the anxiolytic effect of crocin has yet to be determined (Pitsikas et al., 2008 ▶). Besides, numerous experiments indicated that dopamine and glutamate pathways are interconnected (Kvetnansky et al., 2009 ▶; Schiller et al., 2010 ▶). 

Indeed, secretion of glucocorticoid is controlled by a negative feedback which inhibits glutamate receptors (GluR) in hypothalamus, pituitary and especially in the amygdala. Additionally, depending on the degree of stress, the very feedback may not be responsible and lead to a high level of glucocorticoid in blood serum (Yehuda, 2009 ▶). Likewise, PTSD patients revealed a decreased level of cortisol in serum because this feedback did not work properly (Yehuda, 2009 ▶). Hypothalamus-pituitary-axis (HPA) also responds to stress by corticotropin-releasing hormone (CRH) and affects endocrine system (Yehuda 2009 ▶). In fact, corticosterone plays a key role in establishment of contextual fear conditioning and PTSD(Putman et al., 2007 ▶; Knapska , 2009 ▶). Previous studies showed that DBS could modulate neural connections between the anatomical areas (Calleja-Castillo et al., 2013 ▶). According to this fact, DBS might modify this neuro-connection among amygdala and corticotropin-releasing hormone (CRH) neurons in the paraventricular nucleus (PVN) (Petrov et al., 1994 ▶). Consequently, this modification might strongly influence cortisol/corticosterone levels in blood. 

Furthermore, previous studies indicated that aqueous extract of saffron may modifying glutamic receptors and change the mechanism of response to corticosterone by decreasing the expression of *GluR* gene; consequently, corticosterone level increases in blood (Hosseinzadeh, 2010 ▶; Halataei et al., 2011 ▶).

Thus, a combination of these treatments might enhance the corticosterone release in PTSD patients and lead to symptoms alleviation.

 In addition, c-Fos protein expression in the amygdala increases during stressful situations and induces CRH release. CRH has also a considerable role in memory conformation in acute stress and trauma in the amygdala and cortico-limbic area (Petrov et al., 1994 ▶). Abnormal activity of the amygdala in PTSD patients noticeably increases CRH release that consequently desensitizes CRH receptors, and causes cortisol reduction in PTSD patients. 

 Our results indicated that the combination treatment used in this study, decreased c-Fos protein expression, and returned CRH release to normal levels; consequently, receptors responded normally and cortisol hormone rose.

 Also, recent experiment results confirmed the obvious influence of both treatment modules on avoidance and fear behaviors known as cue-related-anxieties which declared in freezing behavior time reduction. Saffron aqueous extract in combination with DBS caused a significant change in general trauma-independent anxiety measured by EPM despite the DBS treatment group in our experiment.

The current experiment findings confirmed that treatment of the BLn amygdala with DBS improved PTSD symptoms such as freezing behavior in male rats. Also, a combination of DBS and saffron resulted in a more marked alleviation of PTSD symptoms such as general anxiety behavior. 
